# Supplementary material for: Orf Virus IL-10 and VEGF-E Act Synergistically to Enhance Healing of Cutaneous Wounds in Mice
Source: J Clin Med. 2020 Apr 11;9(4):1085. doi: 10.3390/jcm9041085 (PMC7231296; doi:10.3390/jcm9041085)
Supplement: Supplementary file 1 [file jcm-09-01085-s001.pdf]

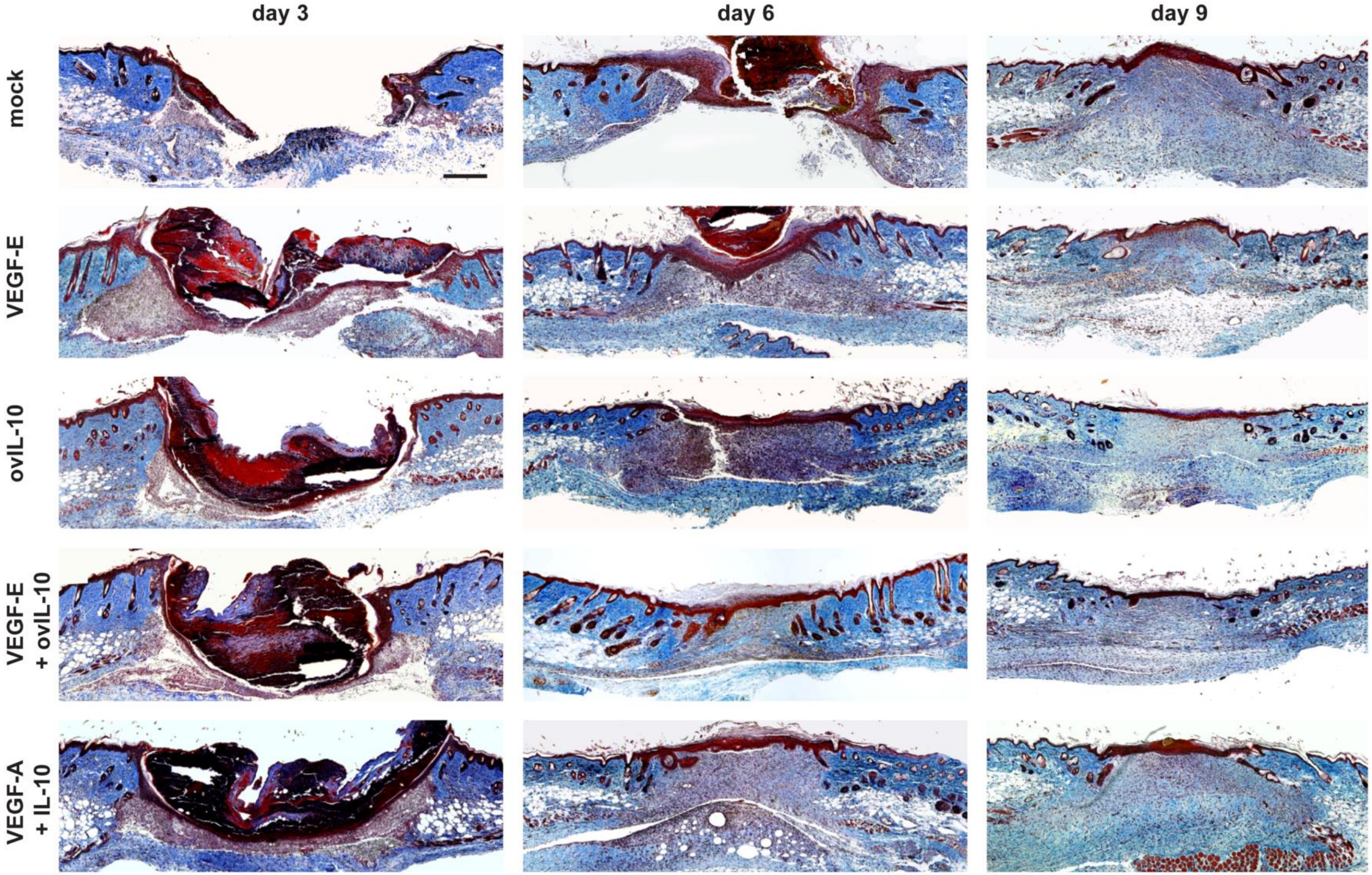

Figure S1. Representative images of MSB trichrome-stained wound sections, taken at 3, 6 and 9 days post-wounding, from mice treated by SC injection with saline (mock), VEGF-E, ovIL-10, VEGF-E and ovIL-10, or VEGF-A and IL-10. Scale bar = 150μm.
